# Negative bias in early and late cognitive processing of coronary heart disease patients with depressive symptoms: an EPR study

**DOI:** 10.1186/s12888-023-05065-4

**Published:** 2023-08-09

**Authors:** Xiaoli Chen, Shupeng Li

**Affiliations:** 1 School of Teacher Education, Weifang University, Weifang City, Shandong Province China; 2 School of Economics and Management, Shandong Vocational College of Information Technology, Weifang City, Shandong Province China

**Keywords:** Coronary heart disease, Depression, Negative bias, Emotional Stroop task, Cognitive impairment

## Abstract

**Background:**

The purpose of this research was to explore the underlying mechanisms of cognitive impairments in patients with coronary heart disease (CHD) who exhibit depressive symptoms. This was accomplished by recording Event-related potentials (ERPs) during the emotional Stroop task, with a specific focus on the temporal dynamics of attentional bias towards various emotional words.

**Methods:**

We selected 17 CHD patients with depressive symptoms and 23 CHD patients without depression using a convenience sampling method from the Affiliated Hospital of Weifang Medical University. Each participant completed an emotional Stroop color-word task, and ERPs were recorded during the task to examine cognitive processing.

**Results:**

CHD patients with depressive symptoms exhibited generally smaller amplitudes of N1, N2, P3 and longer latency of P3 compared to CHD patients without depression. Specifically, the N1 amplitude of negative words was smaller and the P3 amplitude of negative words was larger in the CHD with depressive group compared to the CHD group. Furthermore, within the group of CHD patients with depressive symptoms, negative words elicited a smaller N1 amplitude and larger P3 amplitude compared to positive and neutral words.

**Conclusions:**

CHD patients with depressive symptoms demonstrate decreased attentional resources, leading to cognitive impairments. Notably, significant attentional bias occurs during both early and later stages of cognitive processing. This bias is primarily characterized by an enhanced automatic processing of negative information at the early stage and difficulty disengaging from such information at the later stage. These findings contribute to the existing literature on the cognitive neural mechanisms underlying depression in CHD patients.

## Background

Coronary heart disease (CHD) is a leading cause of mortality in industrialized nations, and several studies have demonstrated a higher prevalence of depression among CHD patients [1–3]. Depression not only increases cardiovascular morbidity and mortality but also negatively impacts the patients' quality of life and medical prognosis [1, 4]. Existing evidence suggests that both CHD patients and individuals with depression experience cognitive impairments. Patients with both CHD and depression are particularly susceptible to cognitive impairment, and higher levels of depression have been linked to reduced cognitive function [5–7]. However, it remains uncertain whether depression or CHD has a greater influence on cognitive impairment.

In a recent study, Chang et al. compared cognitive function across healthy controls, cardiovascular patients, depressed patients, and cardiovascular patients with depression. They found that cardiovascular patients with depression exhibited the lowest cognitive function. Significant differences in executive function were observed only between cardiovascular patients and cardiovascular patients with depression, while no significant differences were found between cardiovascular patients and depressed patients, or between depressed patients and cardiovascular patients with depression [8]. Freiheit et al. also reported that CHD participants with persistent depressive symptoms demonstrated significantly greater declines in attention/executive function, learning/memory, verbal fluency, and global cognition compared to CHD participants without depressive symptoms [9]. These findings suggest that depression has a more substantial impact on cognitive impairment than CHD [10, 11]. However, these studies on cognitive function heavily relied on traditional self-report measures, which may introduce some bias [12]. In recent years, there has been an increasing focus on cognitive neuroscience assessment methodologies, but a research gap remains regarding the cognitive neural mechanisms in CHD patients with depression [13].

The primary aim of this study was to utilize Event-Related Potentials (ERPs) technology to investigate real-time brain activity differences in cognitive function between CHD patients and CHD patients with depression, building on the work of Chang et al. [8]. ERPs offer a more refined and sensitive approach for detecting dynamic cognitive alterations by recording amplitude and latency, providing insights into different stages of cognitive processing with high temporal resolution [14, 15]. Executive function is known to play a crucial role in emotional functioning, and cognitive impairments in this domain may serve as a mechanism underlying depressive symptoms [16, 17]. Extensive research has demonstrated that individuals with depression selectively attend to and process negative stimuli that are congruent with their emotional state. This difficulty in inhibiting attentional processing of negative information contributes to mood and emotional dysregulation [18, 19]. The emotional Stroop task has frequently been employed in clinical studies to investigate electrophysiological abnormalities directly associated with the psychopathology of depressed patients [15, 20, 21]. To the best of our knowledge, there is a dearth of evidence regarding the neurophysiological mechanisms underlying cognitive impairments in CHD patients with depressive symptoms. This knowledge gap underscores the significance and relevance of our study.

Previous ERP studies employing the emotional Stroop paradigm have revealed various cognitive process impairments in individuals with depression. For instance, Dai and Feng found that depressed participants exhibited a smaller N1 amplitude for negative words and a smaller P1 amplitude for positive words, suggesting deficient inhibition for negative words and reduced attention to positive words [22]. Pérez-Edgar and Fox discovered that negative words elicited smaller N1 and N2 amplitudes compared to positive and neutral words [23]. Generally, the N1 component is associated with early perceptual stages of attentional focus on the target and discrimination processing, while the N2 and P3 components reflect more elaborate processing of stimuli in later and higher cognitive stages [24, 25]. The N2 component is considered a marker of conflict detection processes, characterized by a negative deflection around 200 to 300 ms, and it demonstrates enhanced sensitivity to emotional stimuli compared to neutral stimuli [26]. Xue et al. found that individuals with treatment-resistant depression exhibited more negative N2 amplitudes compared to a healthy control group, indicating that depressed patients require greater cognitive resources during the processing of emotional information [27]. The P3 component, a positive deflection occurring approximately 300 to 600 ms after stimulus onset, is a well-known indicator closely associated with conflict resolution processes. P3 amplitude is related to the allocation of cognitive resources and indicates the level of attention drawn by specific stimuli [17, 28, 29]. Many ERP studies have shown that P3 amplitude is typically attenuated in depression compared to healthy individuals, and P3 amplitude is larger for negative words compared to neutral words in the right parietal areas, indicating that depressed patients have inefficient processing during emotional conflict resolution [22, 30, 31].

Therefore, the present study aims to utilize the ERPs method to examine the temporal dynamics of cognitive processing in CHD patients with depression during the performance of the emotional Stroop task. By investigating these aspects, we can gain crucial insights into the neurophysiological mechanisms underlying depression in CHD patients, which can be beneficial for clinical practice and intervention strategies for individuals with CHD and depression. We hypothesize that CHD patients with depression will exhibit impaired interference inhibition, as evidenced by smaller N1 and P3 amplitudes compared to CHD patients without depression. Furthermore, we predict that negative words will elicit smaller N1 and N2 amplitudes but larger P3 amplitude compared to positive and neutral words in CHD patients with depression.

## Methods

### Participants

A priori power analysis was conducted using G*power software to determine the required number of participants. When the medium effect size was set at 0.25, α was 0.05, and power was 0.8, the total number of participants required was approximately 16. A total of 50 participants were recruited for this study, with 25 participants in the CHD group and 25 participants in the CHD with depressive symptoms group. Participant recruitment was based on responses to the Self-rating Depression Scale [32] and Structured Clinical Interview conducted by psychiatrists, adhering to the diagnostic criteria of "single-phase affective disorder, depressive episode" in the Diagnostic and Statistical Manual for Mental Disorders [33]. All participants were CHD patients experiencing their first incidence and were receiving treatment at the Affiliated Hospital of Weifang Medical University. They were right-handed individuals with normal visual acuity and color vision, aged between 40 and 60, and had at least a primary school education level.

The inclusion criteria for the CHD with depressive symptoms group were as follows: 1) meeting DSM-IV criteria for a current episode of depressive disorder, and 2) scoring ≥ 72 on the Self-rating Depression Scale (SDS). Participants were excluded if they had: 1) a current or past DSM-IV diagnosis of bipolar disorder, anxiety disorder, schizoaffective disorder, substance abuse/dependence, or organic psychiatric disorder, 2) active suicidal intent or a history of suicide attempts during the current depressive episode, and 3) organic brain disease, epilepsy, dementia, stroke, or neurodegenerative diseases (e.g., Parkinson's disease). The CHD group needed to fulfill the following criteria: 1) no current or past DSM-IV diagnosis of depressive episode, and 2) scoring ≤ 53 on the SDS [32]. Participants with a history of severe head trauma, alcohol or drug abuse, organic brain disease, or neurological diseases were excluded from the study.

During the course of the experiment, 4 CHD patients with depression withdrew due to physical reasons, and 6 patients were excluded from the analysis due to excessive head movement. The data from the remaining 17 CHD patients with depression (6 women) and 23 CHD patients (9 women) were included in the final analysis. Although the two groups differed significantly in terms of depression scale scores, there were no significant differences in age or years of education (refer to Table 1). Ethical approval for the study was obtained from the Research Ethics Committee of Weifang Medical University, and all participants provided written informed consent prior to the experiment.

**Table 1:** Demographic data of CHD with depressive group and CHD group(M ± SD)

Characteristics	The CHD with depressive group	The CHD group	t	*p*	Cohen’s d	Effect size
SDS	76.86 ± 3.68	30.76 ± 8.75	22.35	< 0.01	6.87	0.96
Age	56.36 ± 6.62	56.29 ± 5.37	0.04	0.97	0.01	< 0.01
Years of Education	9.18 ± 2.42	9.18 ± 2.48	0.01	0.10	< 0.01	< 0.01

### Stimuli

A total of 90 emotional adjectives were selected from the Chinese Affective Words System, based on established norms for valence and arousal [22]. The adjectives were divided into three categories: 30 positive, 30 neutral, and 30 negative. The selection of words was guided by specific criteria for each category. Positive words were chosen to have higher valence (value > 7) and arousal (value > 5). Neutral words were selected to have neutral valence (3 < value < 5) and limited arousal (value < 3). Negative words were chosen to have lower valence (value < 3) and higher arousal (value > 5) [18]. The colors red, green, and blue were used to present the words on a video monitor. All 270 possible combinations of word content and color presentation were presented at the center of a black background. The words were displayed at a distance of 100cm from the participants, resulting in a visual angle of approximately 7.27° × 6.06°. Each trial consisted of one emotional adjective in Song typeface using a 72-point font, presented in one of the three colors. Each color occurred with equal frequency for each type of emotional adjective.

### Study design and procedure

A mixed experimental design was employed, with the factors of group (CHD with depressive group, CHD group) and word valence (positive, neutral, negative). Group served as the between-subject factor, while word valence was the within-subject factor. The dependent variable was the ERP component induced by the participant's recognition of emotional words.

Prior to the start of the experimental tasks, participants completed 24 practice trials that were similar to the main experiment to ensure that they understood the task requirements. The formal experiment was divided into three blocks, with each block containing 90 trials consisting of 30 positive words, 30 neutral words, and 30 negative words, corresponding to the colors of red, green, and blue, respectively. The 270 trials were presented in pseudo-random order within each block. After completing each block, participants took a 10-min break.

Each trial began with the presentation of a central fixation cross that was displayed for 800 ms, followed by the presentation of the emotional words in a randomized order. The stimuli remained on the screen until the participants responded, and any trial where the response was not provided within 1500 ms was excluded from subsequent analysis. Participants were seated in a sound-attenuated and dimly lit cabin and instructed to only identify the color of the words (red, green, blue) while ignoring the meaning of the words. They were instructed to respond by pressing the correspondingly labeled buttons on the keyboard with their left index finger (F key), right index finger (J key), or right middle finger (K key), respectively. Participants were required to respond as accurately and quickly as possible, and the response fingers were counterbalanced across subjects.

### EEG recording and EEG data analysis

The experimental equipment was produced by the American Neuroscan Company. Brain Electroencephalograms (EEGs) were recorded by using 64 sintered Ag/AgCI electrodes located in expanding 10/20 electrode position embedded in an elastic cap recording device [34]. All electrodes were online referenced to the left mastoid and re-referenced offline to the average of both mastoids. In addition, vertical Electrooculogram (EOG) was monitored from electrodes positioned below and above the right eye and horizontal EOG was monitored from the external left and right canthi. The continuous EEG was sampled at a rate of 1000 Hz with a band pass of 0.05–100 Hz, and electrode impedance was maintained below 5 kΩ.

The EEG data were averaged based on combinations of valence (positive, neutral, negative) and group (CHD with depressive symptom, CHD). Offline filtering was applied, using a low-pass filter at 16 Hz (24 dB/octave). The epoch for averaging ranged from -100 ms to 1000 ms relative to the onset of the stimulus. Baseline correction was performed by referencing the data to the activity in the 0–200 ms window before stimulus onset. Trials containing incorrect responses or EOG artifacts (amplitudes exceeding ± 100 μV) were excluded from analysis. For statistical analysis, 18 electrode sites were selected based on previous studies and localization research related to intention. These sites included F3, FZ, F4, FC3, FCZ, FC4, C3, CZ, C4, CP1, CPZ, CP2, P3, PZ, P4, PO3, POZ, PO4 [35, 36].

**Fig. 1:**
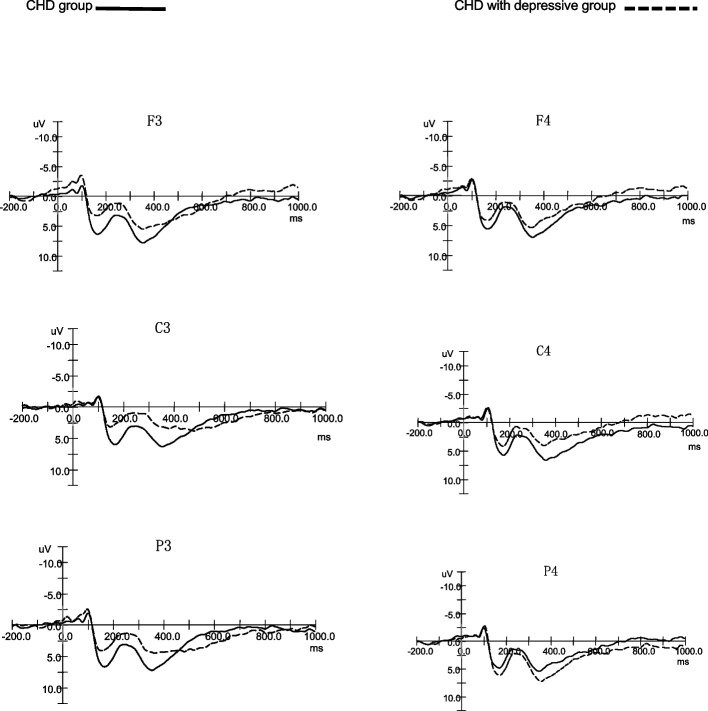
The ERPs of positive words

**Fig. 2:**
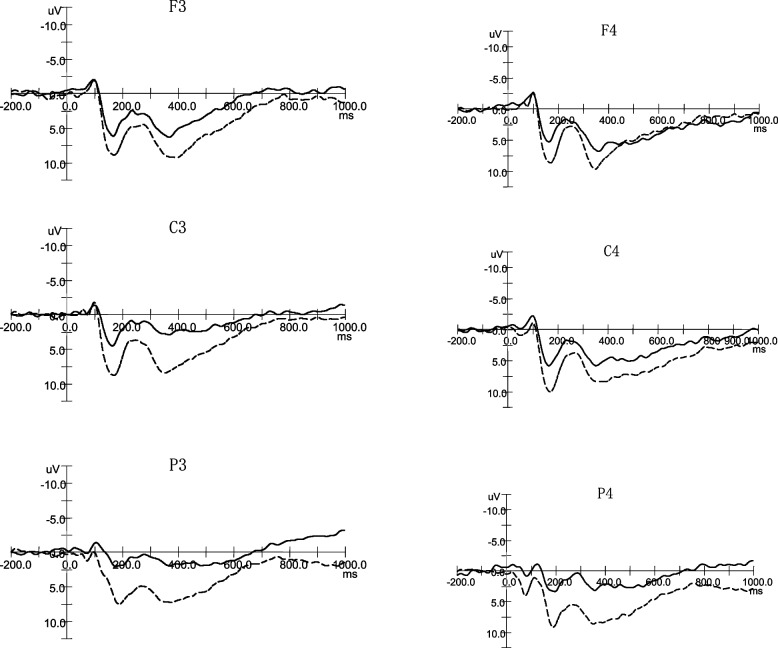
The ERPs of negative words

The peak amplitude and latency of specific ERP components were determined for each electrode site. The N1 component was defined as the largest negative peak occurring within a latency window of 80–120 ms. The N2 component was identified as the largest negativity within the time window of 220–290 ms. The P3 component was characterized as the largest positive peak within a 300–390 ms window. Since the P3 peak might not be easily discernible, the mean offset P300 amplitude was quantified as the mean voltage during this time range [20]. In the study, a repeated measures ANOVA was conducted separately on the amplitude and latency of the N100, N200, and P300 components. The ANOVA included factors of group (CHD with depressive group, CHD group), word valence (positive, neutral, negative), and laterality (left hemisphere, middle line, right hemisphere). The significance level was 0.05, Greenhouse–Geisser corrections were applied when the assumption of sphericity was violated, and Bonferroni tests were used for multiple comparisons. Figures 1 and 2 present the grand average ERPs elicited by positive and negative words in each of the two groups.

## Results

### The amplitude and latency of N100

The ANOVA for N1 amplitude revealed a significant main effect of group, F(1, 37) = 6.45, *p* = 0.01, *η*^*2*^ = 0.03, indicating that the CHD with depressive group had a smaller amplitude compared to the CHD group. There was also a significant interaction between group and word valence, F(2, 74) = 6.23, *p* = 0.002, *η*^*2*^ = 0.05. Further analysis showed that the amplitude of negative words was smaller in the CHD with depressive group compared to the CHD group, F(1, 37) = 15.13, *p* < 0.001. Additionally, only the CHD with depressive group exhibited a smaller amplitude for negative words compared to positive and neutral words, F(2, 37) = 15.39, *p* < 0.001. No significant effects were found for N1 latency (*p*s > 0.05).

### The amplitude and latency of N200

For N2 amplitude, there was a significant main effect of group, F(1, 37) = 6.41, *p* = 0.01, *η*^*2*^ = 0.03, indicating a smaller amplitude in the CHD with depressive group compared to the CHD group. The main effect of hemisphere was also significant, F(2, 37) = 28.66, *p* < 0.001, *η*^*2*^ = 0.20, showing a right-hemisphere dominance. The two-way interaction between word valence and hemisphere was significant, F(4, 74) = 9.43, *p* < 0.001, *η*^*2*^ = 0.04, with further analysis revealing that the amplitude of negative words was larger compared to positive words in the right hemisphere, F(2, 37) = 4.15, *p* = 0.04. No significant differences were observed in the left hemisphere, F(2, 37) = 2.65, *p* = 0.07. No significant effects were found for N2 latency, except for the main effects of hemisphere (longer latency in the left hemisphere), F(1, 37) = 6.12, *p* = 0.003, *η*^*2*^ = 0.03, and word valence (longer latency for negative words), F(1, 37) = 28.10, *p* < 0.001, *η*^*2*^ = 0.11.

### The amplitude and latency of P300

The ANOVA for P3 amplitude yielded significant main effects of group (F(1, 37) = 9.43, *p* = 0.002, *η*^*2*^ = 0.04), word valence (F(2, 37) = 32.94, *p* < 0.001, *η*^*2*^ = 0.22), and hemisphere (F(2, 37) = 9.32, *p* < 0.001, *η*^*2*^ = 0.08). The CHD with depressive group had a smaller amplitude compared to the CHD group. Negative words elicited larger amplitudes compared to positive and neutral words. The largest P3 amplitude was observed at the middle line, and the left hemisphere had a smaller amplitude compared to the right hemisphere. A significant interaction between group and word valence was also found, F(2, 74) = 13.23, *p* < 0.001, *η*^*2*^ = 0.10. Further analysis showed that the CHD with depressive group had a smaller amplitude for positive words (F(1, 37) = 3.92, *p* = 0.05) and a larger amplitude for negative words compared to the CHD group (F(1, 37) = 47.52, *p* < 0.001). Additionally, only the CHD with depressive group exhibited a larger amplitude for negative words compared to positive and neutral words, F(1, 37) = 11.74, *p* < 0.001. The CHD group did not show a significant difference in amplitude among the three word valences, F(1, 37) = 2.51, *p* = 0.08. The analysis of P3 latencies revealed a significant main effect of group, F(1, 37) = 28.25, *p* < 0.001, *η*^*2*^ = 0.11. Specifically, the CHD with depressive group exhibited a delayed P3 compared to the CHD group. Additionally, a significant main effect of word valence was found, F(2, 37) = 3.70, *p* = 0.03, *η*^*2*^ = 0.02. Negative words elicited longer latency compared to positive words. No significant effects were observed for hemisphere or interactions (*p*s > 0.05).

## Discussion

The aim of this study was to investigate cognitive information processing in CHD patients with depression by recording ERPs during the emotional Stroop task. The emotional Stroop task has been widely used to assess biased information processing, while ERPs provide valuable insights into neuropsychological deficits. ERPs are characterized by small voltage fluctuations that reflect real-time brain activity within milliseconds, allowing for the examination of the temporal dynamics of emotional information processing [17, 37, 38]. Early components such as N1 and P1 are influenced by physical stimulus parameters, whereas later components such as N2 and P3 are associated with more complex cognitive processes and specific perceptual or cognitive stages [27, 39, 40].

In the present study, the analysis of N1 amplitude revealed that negative words elicited smaller N1 responses compared to positive and neutral words in the CHD with depressive group. The N1 component is an early component that reflects attentional focus on the target and the discrimination process within that focus of attention [39, 41]. According to the theory of finite computational resources, individuals must selectively process information, allocating more thorough processing to certain stimuli over others [40]. Therefore, these results suggest that biased information processing occurs at an early stage, coupled with limited early attentional resources for negative words in CHD patients with depressive symptoms. Furthermore, the amplitude of negative words was smaller in CHD patients with depressive symptoms compared to CHD patients, indicating reduced attentional resources allocated to negative words in individuals with depressive symptoms. One possible interpretation of this finding is that semantic information processing is automatic, and individuals with CHD and depressive symptoms tend to selectively attend to negative information that aligns with their current mood state, making them more efficient in detecting negative-relevant information compared to other stimuli [18, 23].

Another important ERP component is N2, which is a negative deflection peaking around 200 to 300 ms after the presentation of visual stimuli. The amplitude of N2 is influenced by the difficulty in distinguishing between categories and reflects the processes involved in response selection [42, 43]. In our analysis of N2 amplitude and latency, we found a significant main effect of hemisphere, indicating a right-hemisphere dominance. Additionally, we observed larger N2 amplitudes for negative words compared to positive words specifically in the right hemisphere. It is widely known that the brain has partial lateralization, with the left hemisphere primarily responsible for word processing and the right hemisphere playing a critical role in emotion processing [44]. Some scholars have suggested that emotional words can induce corresponding emotions, and unattended emotional words tend to receive priority in attentional resource competition, leading to attention bias [45]. As a result, negative stimuli can elicit stronger emotional effects in the right hemisphere [46]. These findings were not observed in previous studies, and this discrepancy could be attributed to differences in subjects and stimuli used [18, 22].

The P300 component is one of the most extensively studied ERP components. It typically refers to the waveform observed within a temporal window ranging from 300 to 500 ms after stimulus presentation. The P300 signifies the allocation of attentional resources to an identified stimulus and is interpreted as a precursor to more elaborate processing [41, 47]. In the present study, we found that the CHD with depressive group exhibited smaller P3 amplitudes and longer latency compared to the CHD group. These findings can be interpreted as evidence of attenuated attentional resources and longer evaluation time in the CHD with depressive group. The observed patterns of P300 effects were consistent with the clinical symptoms of CHD patients with depression, such as decreased ability to concentrate, reduced speed of mental processing, and psychomotor retardation [48].

Furthermore, the CHD with depressive group displayed larger amplitudes and longer latency for negative words compared to positive words. This suggests that CHD patients with depressive symptoms exhibit a lack of resource deployment to positive words, while negative words require extended processing resources at a later stage of elaboration. This may be attributed to inhibition deficits towards negative information and difficulties in disengaging from them in CHD patients with depressive symptoms. As a result, more cognitive resources are allocated to negative words, leading to longer processing times for negative words and limited cognitive resources available for positive words [20, 49]. It is possible that the biased attentional processing of negative information in CHD patients with depressive symptoms contributes to difficulties in mood and emotion regulation [19, 50].

The present study has certain limitations that should be acknowledged. Firstly, the participants were selected using a convenience sampling method from depressed patients at the Affiliated Hospital of Weifang Medical University. Therefore, caution should be exercised when generalizing these findings to other regions, as regional differences may influence the conclusions and implications. Secondly, this study represents a preliminary investigation into the cognitive neural mechanisms among coronary heart disease patients with depression, utilizing the emotional Stroop task. Future studies need to be further refined to explore the effect of depression severity on the emotional Stroop task.

## Conclusions

The present study investigated the cognitive processes in CHD patients with depressive symptoms using emotion-word Stroop tasks, which shed light on the attention bias to emotional information. Findings support the notion that cognitive impairments of CHD patients with depressive symptoms occur at both early and later stages. Specifically, these impairments are evident in the heightened automatic processing of negative information during the early stage, as well as the difficulty in disengaging from such information during the late stage. This research adds to the psychocardiology literature and deepens our understanding of the pathogenesis of CHD with depressive symptoms.

## Data Availability

The datasets used or analysed during the current study are available from the corresponding author on reasonable request.

## Abbreviations

CHD: Coronary heart disease
ERPs: Event-related potentials
DSM: Diagnostic and Statistical Manual for Mental Disorders
SDS: Self-rating Depression Scale
EEGs: Electroencephalograms
EOG: Electrooculogram
